# Seizure Management and Prophylaxis Considerations in Patients with Brain Tumors

**DOI:** 10.1007/s11912-023-01410-8

**Published:** 2023-04-18

**Authors:** Nils Stenvågnes Hauff, Anette Storstein

**Affiliations:** Department of Neurology, Haukeland University Hospital, Jonas Lies Vei 65, 5021 Bergen, Norway

**Keywords:** Neurooncology, Brain tumors, Epilepsy, Glioma, Status epilepticus

## Abstract

**Purpose of Review:**

The article gives an overview of the current knowledge in the management of tumor related epilepsy, including systematic reviews and consensus statements as well as recent insight into a potentially more individualized treatment approach.

**Recent Findings:**

Tumor molecular markers as IDH1 mutation and MGMT methylation status may provide future treatment targets. Seizure control should be included as a metric in assessing efficacy of tumor treatment.

**Summary:**

Prophylactic treatment is recommended in all brain tumor patients after the first seizure. Epilepsy has a profound effect on the quality of life in this patient group. The clinician should tailor the choice of seizure prophylactic treatment to the individual patient, with the goal of limiting adverse effects, avoiding interactions and obtaining a high degree of seizure freedom. Status epilepticus is associated with inferior survival and must be treated promptly. A multidisciplinary team should treat patients with brain tumors and epilepsy.

## Introduction

Epileptic seizures are common in patients with brain tumors. Both the seizures and the treatment contribute significantly to the disease burden. The following article provides an overview of the current knowledge in management of brain tumor–related epilepsy. The article will focus on gliomas, which are the most common primary brain tumors in adults. Developments in research may lead to more individualized seizure treatment options for these patients in the future.

## Epidemiology

Malignant and nonmalignant brain CNS tumors are diverse and vary greatly in epidemiology, histological subtypes, clinical characteristics, treatment, and outcome. The incidence of primary brain and other CNS tumors is an estimated 23.8 per 100,000 [1]. Malignant tumors account for 30% of CNS tumors [2]. In the following, we will mainly focus on seizures in gliomas. Gliomas are the most common primary brain tumor entity in adults and represents about 25% of the total tumor incidence and about 80% of the malignant tumors of the CNS [1].

Seizures are common in patients with brain tumors, and both the seizures and the treatment contribute significantly to the burden of disease. Between 25 and 60% of patients with brain tumors develop epilepsy over the course of their disease, and seizure is the presenting symptom in 20–40% of cases [3]. Diffuse gliomas are the most common primary brain tumors in adults, and 40–70% of these patients have one or more epileptic seizures over the course of disease [4].

Seizures have been associated with improved survival, in particular in diffuse glioma. A seizure is often the precipitating event for diagnosis, and epilepsy is more prevalent in IDH1 mutant tumors, which have a better prognosis [4]. Epilepsy is less common in patients with glioblastoma, and is not associated with inferior overall survival [5].

## Etiology

The underlying pathophysiology of brain tumor–related epilepsy (BTRE) remains poorly understood. Multiple factors contribute to BTRE, including speed of tumor growth, location, and tumor histology. There is an inverse relationship between seizure prevalence and tumor growth rate [3], presumably due to slow-growing tumors being more epileptogenic. Tumors involving the frontal, temporal, and parietal cortex are more epileptogenic. In low-grade gliomas [3], lesions involving the left premotor area are more often associated with bilateral tonic-clonic seizures. The pathogenesis of epilepsy is clearly multifactorial [6]; perturbations in an epileptogenic peritumoral zone have been implicated [7].

Epileptogenesis and tumor progression are likely to have a dual relationship. The neuronal hyperexcitability of epilepsy might promote tumor growth [8]. The major excitatory neurotransmitter implicated in seizures, glutamate, also stimulates tumor cell growth and infiltration. The AMPA receptor subtype is of special interest, and AMPA receptor antagonist drugs could have a dual benefit in reducing seizures and tumor growth alike.

Developments in molecular profiling have radically altered the classification of numerous cancer types, brain tumors no exception [9••]. For gliomas, isocitrate dehydrogenase (IDH) 1 and 2 mutations are driver mutations and of major prognostic and therapeutic significance. IDH mutations are much more frequent in gliomas of grades 2 and 3, and IDH non-mutated/wild type (wt) tumors have a markedly worse prognosis, regardless of histological features. Correspondingly, in the most recent WHO classification, the group of glioblastoma tumors now includes IDH wt gliomas of histological grades 2 and 3 with a glioblastoma-like molecular profile.

In line with this, studies of the last few years show that tumor molecular markers are of importance concerning preoperative seizures and post-operative seizure control [4]. Seizures in glioma are more associated with isocitrate dehydrogenase (IDH) 1 and 2 mutated tumors and MGMT methylation status than grade, location, or histopathology [4]. Patients with IDH mutated grade 2 gliomas are much more likely to experience medication-refractory seizures than IDH wt grade 2 patients. Within the group of IDH wt tumors [10], grades 2 and 3 tumors are more likely to present with early seizures, have more seizure days, and require more often polytherapy than IHD wt glioblastomas [11].

The product of the mutated IDH enzyme is the oncometabolite d-2-HG that mimics the action of glutamate at the NMDA receptor, the major excitatory neurotransmitter. This has been proposed as the reason why the IDH1 mutation is associated with more prevalent seizures [12]. However, one recent study found that d-2-HG lead to neuronal spiking and was a potent mTOR activator in neuronal cultures and in tumor tissue [13•, 14••] from patients with IDH mutated tumors. The mTOR signaling pathway is a driver of epilepsy in tuberous sclerosis complex and focal cortical dysplasias [14••]. The mTOR inhibitor rapamycin is shown to reduce neuronal activation and suppress seizures in models. These findings potentially opens new therapeutic options for patients with seizures and IDH mutant gliomas [14••].

## Status Epilepticus

Between 3 and 12% of all adult status epilepticus cases are caused by brain tumors [15]. Mortality attributed to status epilepticus, persistent or recurrent seizures within a 5 min interval, is nearly three times higher in patients with brain tumors than in epilepsy patients generally. The appearance of tumor-associated status epilepticus (TASE) can indicate tumor progression [5, 15]. In half of the patients with glioma, status epilepticus is due to other factors such as non-adherence to medication or intercurrent conditions, for instance infections or electrolyte disturbances [16]. A study from 2021 showed that the semiology of TASE was more commonly complex focal (74%) and less commonly generalized convulsive (17%) [5].

Status epilepticus in patients with brain tumors should be treated promptly with first line therapy (diazepam, midazolam), and second line therapy (levetiracetam, phenytoin, valproic acid) whenever the seizures do not respond.

While it is not clear that epilepsy in general is associated with inferior survival, status epilepticus is clearly associated with inferior survival in patients with malignant brain tumors [5].

## Primary Seizure Prophylaxis

Recent studies have shown that patients with newly diagnosed brain tumors who have not ever experienced seizures do not benefit from antiseizure medication (ASM) [11]. Primary prophylaxis is unlikely to be effective in increasing progression-free survival or reducing the frequency of a first seizure within 6 months from diagnosis. A consensus statement thus discourages the use of primary prophylactic use of ASM [17••].

## Secondary Seizure Prophylaxis

ASM is recommended for all brain tumor patients who have experienced at least one seizure. According to the International League Against Epilepsy (ILAE) guidelines, patients with a structural abnormality that increases the risk of new seizures fulfill the criteria for epilepsy after one episode only [18]. In general, lamotrigine and valproic acid are regarded as the most efficient drugs in focal epilepsies, and have proved to be efficient in patients with brain tumors [22]. So far, histology, location, and grading do not directly influence the choice of ASM. However, in BTRE, additional considerations are required with respect to the multifactorial etiology, oncological therapy, future disease progression, and the total burden of disease in these patients. Rapid titration regimens and availability of intravenous administration are often advantageous in patients waiting for tumor surgery or with a short life expectancy [19•], and in patients with dysphagia. Levetiracetam and lacosamide are advantageous due to the ability to rapidly titrate these medications, intravenous and for levetiracetam liquid formulations, and widespread availability.

Brain tumor patients are vulnerable to neurological adverse effects of ASM, especially cognitive decline, depression, and anxiety [20••], although other factors contribute considerably in this population. Cognitive adverse effects are most common for first generation ASM. Enzymatic effects on oncological therapy and steroids are another concern. Thus, first-generation ASM are seldom used for brain tumor patients. Valproic acid is effective but may cause hematological toxicity, and potentially in a synergistic fashion in patients receiving chemotherapy.

Levetiracetam is the present drug of choice for many neuro-oncologists [21]. In a recent publication, levetiracetam was more efficient than valproic acid, although side effects were comparable [22]. However, levetiracetam is prone to cause psychiatric side effects. In one study, patients with glioma on levetiracetam were more likely to use anti-anxiolytic drugs [23], although this is not a consistent finding. On the other hand, some AM have beneficial side effects such as mood stabilization and anxiolytic effects. Thus, the clinician should take a careful psychiatric history prior to deciding on the most appropriate choice of ASM, and make a point to check in on the psychiatric welfare of patients on ASMs that may impact mood.

A recent systematic review finds levetiracetam, pregabalin, and phenytoin to be the most efficient drugs in the monotherapy setting [24]. Lacosamide as monotherapy [25] has been investigated in several studies, the largest of which reported seizure freedom for 65% at 3 months and 55% at 6 months. There are relatively few studies on lamotrigine in BTRE. In a recent study, lamotrigine and lacosamide were equally effective in reducing seizure frequency after one year of observation [26]. Lamotrigine metabolism may be influenced by drugs with C-P450 effects, and the slow titration rate and required oral administration is a potential disadvantage in patients with brain tumors.

About a third of patients have recurrent seizures on monotherapy, and add-on therapy is indicated. Patients with BTRE and polytherapy often suffer significantly more side effects and experience a reduction in quality of life [27], whereas the seizures had a lesser impact. Thus, the clinician must weigh the benefits and drawbacks for the individual patient when polytherapy is considered. Some patients with stereotype partial focal seizures may prefer recurrent seizures over increased side effects from medication, while patients with more generalized seizures or prolonged postictal phases may tolerate more side effects to remain seizure free.

No single ASM seems superior to others as add-on therapy [28]. Brivarecatam as add-on therapy has been explored in one small retrospective study [29], with a monthly seizure reduction from 7 to 2. Perampanel is a non-competitive AMPA antagonist, and is of special interest due to the potential anti-tumor benefits. Patients (57%) treated with add-on perampanel in several small studies achieved seizure freedom [30]. Lacosamide was effective and well tolerated in two prospective studies [31, 32]. Valproic acid and lamotrigine are other add-on options [33].

A percentage of glioma patients have a drug-refractory epilepsy that requires a second add-on drug (or third ASM), in particular patients with IDH1 mutated glioma [10].

Patients whose epilepsy develops to drug-refractory seizures should be monitored closely for tumor progression as, in our experience, re-emergence of seizures sometimes herald radiological findings of tumor growth.

Withdrawal of ASM in glioma patients is a debated issue where no consensus exists. In patients with gliomas, studies have found that the majority (71%) of seizure relapses occur in the first 6 months after withdrawal [34]. Whether discontinuation of ASM is relevant at all depends upon a number of factors, including residual tumor, oncological prognosis, and social consequences of recurrent seizures [35]. Patients with a prior history of frequent seizures or epileptogenic discharges on EEG are likely to be at high risk of relapse after withdrawal and should probably continue long-term therapy [36].

Concerning emerging therapies in glioma epilepsy, the IDH1 mutation may represent a new therapeutic target. One case study reports that the IDH1 inhibitor ivosidenib improved drug-refractory seizures in a patient with an IDH mutated oligodendroglioma [37]. As discussed above, the epileptogenic action of the d-2-HG oncometabolite and its ability to activate the mTOR pathway open up the potential use of mTOR inhibitors as rapamycin, everolimus, and temserolimus for patients with IDH1 mutated tumors and drug refractory epilepsy [38].

## Effects of Surgery on Seizures

Functional mapping–based surgery is today the first choice therapy in diffuse low grade gliomas and enables improvement of both overall survival and quality of life [39]. Tumor resection significantly impacts postoperative seizure control, but the precise extent of resection required to translate to improved survival is a topic of discussion [40]. The extent of resection is a significant predictor of seizure control and quality of life [40]. Recent studies show that higher percent of tumor resection independently predicts good postoperative seizure control. Different thresholds of ≥80 and ≥91% have been suggested [39, 40]. Total or near-total resections of diffuse low-grade gliomas are however often difficult to achieve due to the infiltrative nature of the tumors which may invade functionally critical structures [40].

With improvements in neurosurgical treatments, patients have access to increasingly advanced treatment, which result in better survival outcome and seizure control. An instance of this are lesions in the region of the insular cortex which historically have been considered too high risk to approach due to the surrounding critical structures. New results show that excision of high-grade gliomas in the insula can be safe and result in good postoperative seizure control [41].

## Effects of Oncological Treatment on Seizures

Oncological therapy (chemotherapy, radiotherapy) may lead to better seizure control in patients with gliomas [42, 43]. In particular, the European Organisation for Research and Treatment of Cancer (EORTC) study showed that postoperative radiotherapy improved seizure control in patients with low-grade glioma [44]. Clinical trials have often not focused on seizure control as an outcome measure. The effect of antitumor treatment on seizure control is, however, of value in the assessment of tumor response [42, 43]. Currently, assessment criteria for tumor response are largely based on MRI findings. In general, seizure reduction appears to be more common in patients with radiological response. Radiological assessment can however be difficult and may not reflect the clinical benefit of the treatment given, so using both radiographic and seizure outcome measures may improve accurate assessment of tumor behavior.

## Non-epileptic Attack Disorder in Patients with BTRE

In patients with epilepsy, the prevalence of non-epileptic attack disorder (NEAD) is as high as 40% [46]. The prevalence of non-epileptic attacks in patients with BTRE is unclear. The diagnosis and treatment of NEAD are challenging in all patients with epilepsy, and the coexistence of a brain tumor may make it even harder for clinicians to recognize the diagnosis. Unrecognized NEAD in patients with BTRE may lead to unnecessary treatment and could delay or disturb oncological treatment. A case study published in April 2022 [46] advised clinicians to always consider the possibility of NEAD in BTRE patients with escalating symptoms, especially in cases where imaging appears to show disease stability.

## Conclusions

Epilepsy is common and a major cause of poor quality of life in patients with brain tumors. While seizure control is usually not the primary reason for maximum surgical resection, radiation therapy, or chemotherapy, it is important that clinicians follow seizure type, frequency, and severity. Seizures and seizure outcomes are to an increasing recognized as another way to monitor tumor activity, and are being used as outcome measures in clinical trials [47]. Seizure control is also widely proposed as a new metric in assessing efficacy of tumor treatment [42, 45, 47]. As survival and progression-free survival improve, the focus of patients and caregivers increases on others factors influencing quality of life that can be impacted by seizure treatment, frequency, and type, such as the ability to work, drive a car, or even get pregnant.

With the emerging focus on the mechanisms of tumor molecular markers such as IDH1 mutation and MGMT methylation status, new treatment targets and better evidence to guide clinical decision making is a hopeful future [4]. Treating epilepsy in patients with brain tumors is already a specialized field and should be tailored to the needs and risk factors of the individual patient. Optimal follow-up of patients with BTRE includes a multidisciplinary team of oncologists, neurologist, neurosurgeons, neurophysiologists, neuropsychologists, specialized nurses, and social workers.
